# *HLA-*associated polymorphisms in the HIV-2 capsid highlight key differences between HIV-1 and HIV-2 immune adaptation

**DOI:** 10.1097/QAD.0000000000001753

**Published:** 2018-03-21

**Authors:** Thushan I. de Silva, Aleksandra Leligdowicz, Jonathan Carlson, Miguel Garcia-Knight, Clayton Onyango, Nicholas Miller, Louis-Marie Yindom, Stephane Hué, Assan Jaye, Tao Dong, Matthew Cotten, Sarah L. Rowland-Jones

**Affiliations:** a Medical Research Council Unit The Gambia, Banjul, The Gambia; b Department of Medicine, Wright Fleming Institute, St. Mary's Campus, Norfolk Place, Imperial College London, London; c Weatherall Institute of Molecular Medicine, MRC Human Immunology Unit, John Radcliffe Hospital, Oxford, UK; d Microsoft Research, Redmond, Washington, USA; e NDM Research Building, Nuffield Department of Clinical Medicine, University of Oxford, Oxford; f London School of Hygiene and Tropical Medicine, London, UK.

**Keywords:** cytotoxic T-lymphocyte, HIV-2, *HLA*

## Abstract

**Objective:**

HIV-1 frequently adapts in response to immune pressure from cytotoxic T-lymphocytes (CTL). Many HIV-2 infected individuals have robust capsid-specific CTL responses associated with viral control. Despite this CTL pressure, adaptive changes in this key immunogenic HIV-2 protein have not previously been described. We sought to compare selective pressure on HIV-1 and HIV-2 capsids and identify HLA-associated viral polymorphisms in HIV-2.

**Design and methods:**

Bioinformatic algorithms to identify sites under positive and negative selective pressure and a statistical model of evolution to identify *HLA-*associated polymorphisms in HIV-2 was applied to sequences from a community cohort in Guinea-Bissau. IFN-γ ELISpots were used to compare T-cell responses to wild-type and variant epitopes.

**Results:**

We identified greater purifying selection and less sites under positive selective pressure in HIV-2 compared with HIV-1. Five HIV-2 codons with *HLA-*associated polymorphisms were detected all within or around known or predicted CTL epitopes. One site was within the *HLA*-B58 SuperType (ST)-restricted epitope (TSTVEEQIQW), the HIV-2 equivalent of the HIV-1 TW10 epitope. In contrast to HIV-1, where a T→N mutation at position 3 is associated with resulting loss of CTL control, an E→D mutation at position 5 was observed in HIV-2. Robust CTL responses to the variant HIV-2 epitope were seen, suggesting that HIV-2 adaptation may be at the level of T-cell receptor recognition.

**Conclusion:**

Greater constraints on evolution may exist in HIV-2, resulting in more purifying selection and different immune adaptation pathways in HIV-1 and HIV-2 capsids. This may allow CTL responses to persist in HIV-2.

## Abstract 2

Supplemental Digital Content is available in the text

## Introduction

HIV-specific cytotoxic T-lymphocyte (CTL) responses are thought to play an important role in HIV-1 control [1–4]. A hallmark of HIV-1 evolution, however, is the rapid appearance of mutations within CTL epitopes, leading to loss of CTL recognition and immune control [5]. HIV-2 differs from HIV-1 in that a substantial proportion of infected people maintain undetectable plasma viral loads for decades with no signs of immunodeficiency. Many others have viral loads 30-fold lower than HIV-1 at equivalent disease stages [6–9]. We have previously demonstrated a strong correlation between the presence of high frequency HIV-2 Gag-specific CTLs and viral control [10–12]. As HIV-2 is able to generate resistance mutations akin to HIV-1 under antiretroviral pressure [13], HIV-2 should also have the capacity to adapt to immune responses similar to HIV-1.

Establishing similarities and differences between HIV-1 and HIV-2 immune evasion strategies has the potential to enhance our understanding of HIV pathogenesis. HIV-1 p24 and HIV-2 p26 represent the two major CTL-targeted proteins in these viruses. Here, we provide the first comparison of selective pressure in HIV-1 p24 and HIV-2 p26 capsid sequences from a community cohort in Guinea-Bissau, along with *HLA-*associated viral polymorphisms in HIV-2, which may represent CTL-driven adaptive changes.

## Methods

### Study participants

Antiretroviral therapy (ART)-naïve HIV-1 and HIV-2 mono-infected individuals from Caió, Guinea-Bissau were recruited following written informed consent during serosurveys and case–control studies conducted in this rural community cohort over almost three decades [14]. CD4 and HIV-2 plasma viral load quantification [using an in-house reverse transcriptase (RT)-PCR assay] were performed as previously described [7,12]. Viral loads were available in 75/86 and CD4^+^ counts in 72/86 of HIV-2-infected individuals. Median (range) viral load was 275 (<100–283 542) and CD4^+^ T-cell count 547.5/mm^3^ (100–1705). Thirty of seventy-five (40%) had a viral load of less than 100 copies/ml, which is broadly reflective of the wider cohort [15]. *HLA* class I genotyping on HIV-2-infected patients was performed during a previous study using sequencing [16,17]. Ethical approval was provided by the joint MRC/Gambia Government Ethics Committee, Guinea-Bissau Ministry of Health and the Oxford Tropical Research Ethics Committee (OXTREC), United Kingdom.

### Amplification and sequencing of HIV-1 and HIV-2 capsids

Plasma samples from 55 CRF02_AG HIV-1-infected individuals were used to generate p24 capsid sequences using previously described methods [18] (Supplementary methods). Eighty-six HIV-2 p26 capsid sequences were used in this study: 85 previously generated sequences (GenBank accession numbers GQ48544–GQ485550 and JX570541–JX570562) and 1 new sequence were generated using the same methodology [19].

### Sequence analysis and tests for codon selection

Sites under positive and negative selection in HIV-1 (231 codons) and HIV-2 (230 codons) were identified by comparison of synonymous (d*S,* no amino acid change) and nonsynonymous (d*N,* amino acid change) substitution rates using three different methods in the Datamonkey web-server [20]: single-likelihood ancestor counting (SLAC), fixed-effects likelihood (FEL) and fast unbiased Bayesian approximation (FUBAR) [21,22] (Supplementary methods). A *P*-value cut-off of 0.05 (SLAC and FEL) and posterior probability of 0.95 (FUBAR) was used to define significant positive or negative selection at a codon.

### Identification of *HLA*-associated HIV-2 viral polymorphisms

*HLA-*associated HIV-2 viral polymorphisms were identified using a phylogenetically corrected logistic regression, used extensively for identifying *HLA*-associated HIV-1 viral polymorphisms [23]. This method corrects for phylogenic relatedness, *HLA*-linkage disequilibrium and codon covariation. Class I *HLA* types from 73 HIV-2-infected adults were available for this analysis. Separate statistical tests were constructed for each *HLA*-amino acid pair, limited to *HLA* alleles and amino acids that were observed in at least 5 and at most 68 individuals. To correct for multiple comparisons, we used a 20% false discovery rate (threshold *P* < 0.0008). Epitope predictions were made by scanning the candidate sequence for peptides of 8–11 amino acids, with each prediction made using the supplied *HLA* (2% prior probability distribution).

### IFN-γ enzyme-linked immunosorbent spot

Cryopreserved peripheral blood mononuclear cells (PBMCs) from HIV-2-infected patients were used in ex-vivo IFN-γ enzyme-linked immunosorbent spot (ELISpot) assays as previously described [12], to quantify responses to wild type and variant epitopes (Supplementary methods).

## Results

### Fewer adaptive changes are present in HIV-2 p26 compared with HIV-1 p24

Using three different algorithms [21,22], we evaluated selective pressure evident across the HIV-1 p24 and HIV-2 p26 capsids. In all analyses, there were more sites under positive selective pressure in HIV-1 p24 (6 vs. 2 in SLAC, 8 vs. 2 in FEL and 12 vs. 2 in FUBAR analysis) and more sites under negative selective pressure in HIV-2 p26 (139 vs. 61 in SLAC, 157 vs. 82 in FEL and 151 vs. 131 in FUBAR analysis); Chi-squared *P* < 0.0001, *P* <0.0001 and *P* = 0.0096, respectively, for the three algorithms. A significantly higher mean d*N/*d*S* ratio (95% confidence interval) of 0.249 (0.223–0.277) in HIV-1 compared with 0.099 (0.099–0.110) in HIV-2 (*P* < 0.0001) confirmed the greater purifying selection seen in HIV-2 (i.e. selective purging of deleterious alleles).

**Fig. 1:**
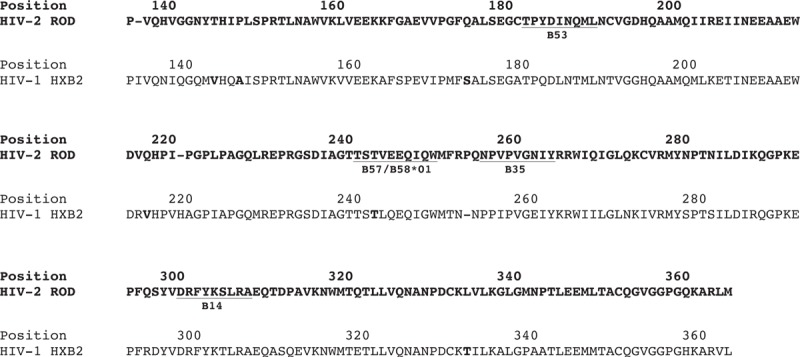
Alignment of HIV-2 ROD (M15390) and HIV-1 HXB2 (K03455) capsid sequences highlighting amino acid positions under positive selection in the Caió population.

We further characterized the sites under positive selective pressure in HIV-1 p24 and HIV-2 p26 (Fig. 1, Supplementary Tables 1 and 2). The two sites under positive selection in HIV-2 (identified by all three algorithms) are not within any known HIV-2 CTL epitope regions, although are in regions flanking *B*^*∗*^*35* and *B*^*∗*^*14-*restricted epitopes, respectively (Fig. 1). Changes in flanking residues can affect epitope processing and presentation, therefore, influencing CTL response [24].

Sites under significant positive selection in HIV-1 CRF02_AG (*n* = 6, identified by all four algorithms SLAC, FEL, FUBAR and REL) and HIV-2 group A (*n* = 2, identified by all three algorithms SLAC, FEL and FUBAR) capsids are highlighted by grey shading. REL was not performed on the HIV-2 set because of alignment size restrictions. Numbering corresponds to Gag positions in ROD and HXB2. Known cytotoxic T-lymphocyte epitopes in the HIV-2 p26 capsid restricted by *HLA* Class I B alleles (common in the Caió population) are underlined. FEL, fixed-effects likelihood; FUBAR, fast unbiased Bayesian approximation; SLAC, single-likelihood ancestor counting and REL.

Three of the four currently known HIV-2 CTL epitopes (restricted by *HLA*-*B*^*∗*^*14*, *B*^*∗*^*3501*, *B*^*∗*^*5301*[12]) were conserved in 96.4, 82.6 and 97.7% of HIV-2 sequences, respectively, despite robust ELISpot responses against these epitopes in *HLA*-*B*^*∗*^*14*, *B*^*∗*^*3501* and *B*^*∗*^*5301*-positive individuals (Supplementary Table 3). This may indicate constraints against evolution within these epitopes, thereby forcing evolutionary change in flanking regions. In contrast, all six HIV-1 p24 sites under positive selective pressure (identified by all three algorithms) lie within putative CTL epitopes restricted by *HLA* alleles common in Caió (Supplementary Table 3), including the well described T242N mutation in the *HLA-B*^*∗*^*57/B*^*∗*^*5801*-restricted TW10 epitope (TSTLQEQIGW) [25,26].

### Identification of a potential *HLA*-associated HIV-2 p26 polymorphism within a known *HLA-B*^*∗*^*5801*-restricted cytotoxic T-lymphocyte epitope

We then identified five associations between *HLA* alleles and polymorphisms in HIV-2 p26 using a previously described statistical model [23] (Table 1). Position 254 (associated with *HLA*-*B*^*∗*^*35*) is immediately upstream of the *HLA-B*^*∗*^*3501*-restricted epitope NPVPVGNIY and position 245 (associated with *HLA-B58* ST) lies within the known *B*^*∗*^*57/B*^*∗*^*5801*-restricted TW10-like HIV-2 epitope TSTV**E**EQIQW (Fig. 1). Positions 256, 294 and 313 were all within or in regions flanking predicted epitopes restricted by the identified *HLA* types (Table 1). The glutamic acid (E) to aspartic acid (D) change at position 245 was observed in 65% of *HLA-B58* ST-positive compared with 32% of *HLA-B58* ST-negative individuals. No HIV-2 sequences had evidence of the HIV-1 equivalent T→N mutation at position 3 (i.e. T242N).

**Table 1:** Potential sites of *HLA*-mediated selection pressure and codon evolution in HIV-2 p26 as predicted by a phylogenetic dependency network analysis.

HIV-2 Gag codon	Amino acid	*HLA* association	Direction^b^	*P* value	*q* value^c^	Predicted epitope around codon associated with relevant *HLA* type^d^	Prediction probability (%)
245	D	B58_ST^a^	Adapted	0.0002	0.0684	STV**D**EQIQW^e^	94
245	E	B58_ST	Nonadapted	6.73E-05	0.0309	STV**E**EQIQW^e^	92
254	A	B35	Nonadapted	0.0002	0.1030	FR**A**QNPVPVGNIYRRW^f^	71
254	P	B35	Adapted	0.0001	0.0336	FR**P**QNPVPVGNIYRRW^f^	71
254	P	C08	Adapted	8.89E-05	0.0336	WMFR**P**QNPVPV	53
256	V	A03_ST^a^	Adapted	0.000749	0.1721	NP**V**PVGNIYRR	51
256	I	A03_ST	Nonadapted	0.000749	0.1721	NP**I**PVGNIYRR	57
294	S	C0401	Nonadapted	2.79E-07	0.0002	E**S**FQSYVDRFYKSLRA	60
294	P	C0401	Adapted	1.10E-07	8.32E-05	E**P**FQSYVDRFYKSLRA	60
313	A	B5801	Adapted	4.93E-08	5.66E-05	QTD**A**AVKNW	66
313	P	B5801	Nonadapted	1.79E-10	4.11E-07	QTD**P**AVKNW	65

^a^ST, SuperType (15).

^b^Adapted indicates that reported amino acid is the putative adaption at that site (i.e. ‘escaped’ variant). Nonadapted indicates that reported amino acid is putatively susceptible to escape (i.e. ‘reversion’).

^c^Estimation of false-discovery rate for each association, that is, *q* value of 0.05 = 5% false discovery rate.

^d^Wherever codon is in flanking region, epitope is underlined. Codon is shown in bold. Epitope predictions are made by scanning the candidate sequence for peptide lengths of 8–11 amino acids. Only peptides within three amino acids of the associated codon are considered.

^e^The 10-mer TSTVEEQIQW has been previously identified as a B58_ST-restricted epitope via functional assays. The prediction algorithm used identifies the 9-mer STVDEQIQW as an optimal B58_ST-restricted epitope.

^f^NPVPVGNIY is a known B^*^35-restricted epitope.

### Robust T-cell responses to the HIV-2 TW10-like wildtype and mutant epitopes despite the presence of the E245D polymorphism in *HLA B*^*∗*^*5801*-positive individuals

T242N escape within the HIV-1 TW10 epitope occurs early after infection, impacts *HLA* binding, leading to loss of CTL recognition and carries a fitness cost overcome by compensatory mutations [25,26]. As robust CTL responses are found in HIV-2-infected individuals decades after infection [10,12], we examined IFN-γ ELISpot responses to both wildtype HIV-2 (TSTV**E**EQIQW) and E245D variant (TSTV**D**EQIQW) peptides. ELISpot responses to both peptide variants were observed in almost all individuals (Supplementary Table 4), including robust responses to the E245D mutant peptide. In all but one individual, the response was stronger against the peptide that matched the individual's autologous virus sequence, suggesting that the T-cell response in these individuals could adapt to overcome this particular CTL epitope polymorphism in HIV-2. In three donors where sufficient PBMCs were available to test, none had cross-reactive responses to HIV-1 TW10 (Supplementary Table 4).

## Discussion

We report the first analysis of *HLA*-associated viral polymorphisms in HIV-2 p26, including a codon substitution within a known immunodominant *HLA-B*^*∗*^*5801*-restricted epitope. This may represent CTL-driven adaptation by HIV-2 and allows direct comparison with what is known about the equivalent *HLA-B57/B*^*∗*^*5801*-restricted epitope in HIV-1. In contrast to HIV-1, wherever a mutation at position 3 of the epitope (T242N) occurs in 63–93% of *HLA*-*B*^*∗*^*5801*-positive individuals [25], a mutation at position 5 (E245D) is found in 65% of *HLA-B58* ST-positive patients. The HIV-1 TW10 epitope lies within a region essential for capsid formation [27] and residue 242 is thought to be critical to stabilizing the electrostatic charge along helix 6 [26]. A T242N mutation reduces this stabilizing effect [26], consistent with viable virus with significantly reduced fitness. It is possible that for HIV-2, with much lower in-vivo viral titres than HIV-1, the fitness costs of such a mutation are too severe, leading to an alternative pathway of immune adaptation. Further functional studies are required to explore this hypothesis.

The E→D mutation found in HIV-2 replaces one hydrophilic, negatively charged, amino acid with another. In contrast to HIV-1 where T242N escape usually results in loss of immune control, robust CTL responses are generated against this HIV-2 mutant. The absence of E245D mutant responses in one participant (B58_8, Supplementary Table 4) suggests that these E245D variant responses do not simply represent cross-reactive CTLs. The presence of CTLs specific to both variants in most individuals may reflect low-level persistence of epitope variants in the viral population not detected by bulk PCR sequencing used in this study. These data also imply that this potential HIV-2 immune adaptation is at the level of T-cell receptor recognition of the peptide–MHC complex, as peptide processing and MHC-epitope binding of E245D variants are presumably still maintained.

We also describe the first comparison of selective pressure on HIV-1 and HIV-2 capsids, finding greater purifying selective pressure in HIV-2. We reported similar findings in HIV-2 *env*, where relative conservation of this highly variable gene is seen despite high magnitude autologous neutralizing antibody titres [28]. Although one explanation for the lower adaptive changes in HIV-2 is lower viral replication compared with HIV-1, previous studies have demonstrated that evolutionary rates are equivalent if not faster in advanced HIV-2 than in HIV-1 infection [29]. Furthermore, the emergence of ART-driven resistance mutations in HIV-2 shows that at least in the reverse transcriptase and protease genes, viral escape can readily occur [13].

A key limitation of our study is the lack of longitudinal data following individual patients from acute HIV-2 infection to demonstrate CTL-driven escape, shown extensively in HIV-1 [1–5]. Although such a study would add considerable insight, the reducing incidence of HIV-2 infection in West Africa, on a background of vastly lower transmission rates than HIV-1, makes this challenging [14,30]. Acute HIV-2 infection is difficult to identify, and therefore, rarely described. As an indirect way of investigating the issue of HIV-2 immune adaptation, we have, therefore, utilized a statistical model of evolution, well validated in HIV-1 cohorts [23,31]. The significant proportion of HIV-2-infected individuals with low viral loads make generating sequence data challenging (approximately 50% success if viral load <100 copies/ml [19]) and could lead to a biased dataset whenever compared with HIV-1. Nevertheless, our dataset represents the largest collection of sequence data generated from HIV-2-infected individuals with viral load less than 100 copies/ml to date [19].

In conclusion, we provide the first evidence of adaptive changes in the HIV-2 capsid. Our data highlight fundamental differences in immune adaptation between HIV-1 and HIV-2, suggesting that HIV-2 evolution may be limited in this region. Further functional studies are required to characterize the polymorphisms identified in HIV-2, validate our findings and explore whether this characteristic explains why robust immune responses can persist in HIV-2-infected individuals for many years. This in turn may underpin the diverse outcomes seen in HIV-1 and HIV-2 infections, providing a crucial clue to the yet unsolved conundrum of the relatively attenuated nature of most HIV-2 infections.

## Acknowledgements

We thank Tim Vincent and the Caió team, as well as the Caió cohort participants, for their invaluable contribution to this study.

### Conflicts of interest

T.I.d.S. was funded at the time of work by an UK Medical Research Council Clinical Research Training Fellowship (G0701313). None of the authors have any competing interests in the manuscript.

Author contributions: T.I.d.S. and A.L. designed the study, conducted experiments, undertook data analysis and wrote the manuscript. J.C. and S.H. undertook data analysis and contributed to manuscript preparation. M.G.K., C.O. and N.M. conducted experiments and contributed to manuscript preparation. A.J., T.D., M.C. and S.L.R.J. designed the study and contributed to manuscript preparation.

## Supplementary Material

Supplemental Digital Content
