# Supplementary material for: HLA-associated polymorphisms in the HIV-2 capsid highlight key differences between HIV-1 and HIV-2 immune adaptation
Source: AIDS. 2018 Mar 21;32(6):709–14. doi: 10.1097/QAD.0000000000001753 (PMC5895130; doi:10.1097/QAD.0000000000001753)
Supplement: Supplemental Digital Content [file aids-32-709-s001.docx]

**Supplementary methods**

*Amplification and sequencing of HIV-1 and HIV-2 capsids*

HIV-1 capsid amplification: Viral RNA was extracted from 200μl of plasma using the QIAamp Ultrasens Viral RNA Extraction Kit (Qiagen) according to manufacturer’s instructions. First round RT-PCR was undertaken using the Titan one Tube System (Roche Applied Science) and primers MO42 (5’-TAGTATGGGCAAGCAGGGAG-3’) and MO44 (5’- TGCCAAAGAGTGATTTGAGGG-3’). Nested PCR was carried out using the first-round product and primers MO43 (5’-TGYGTRCATCAAARGATAGA-3’) and MO45 (5’-CCCCTTGYTGGAAGGCCA-3’).

HIV-1 and HIV-2 sequencing:All HIV-1 and HIV-2 capsids were sequenced by Macrogen^[1]^ using the BigDye Terminator v3.1 cycle sequencing kit and an ABI3730XL sequencer. All new HIV-1 and HIV-2 sequences were submitted to GenBank under accession numbers MG604238 – MG604293.

*Tests for codon selection:*

Estimations were carried out using codon models of evolution selected by a genetic algorithm^[2]^ on the Datamonkey suite: the Hasegawa-Kishino-Yano 85 model for HIV-1 p24 and general-time reversible model for HIV-2 p26 sequences were selected by this algorithm as the best-fitting models for the two datasets. All alignments were tested for recombination using the GARD tool^[3]^. A single recombination breakpoint in each of the HIV-1 and HIV-2 alignments was identified and therefore multiple partition datasets were used for all analyses of selection.

*IFN-*γ*ELISpot*

10^5^ PBMCs were added per well of a 96-well MultiScreen filter plate (Millipore) coated with 15μg/ml of anti-IFN-γ monoclonal antibody (1-DIK, MABTECH). Cells were stimulated for 16 hours at 37˚C, 5% CO_2_ with wild type or variant epitopes at a final concentration of 2µg/ml. Mean spot values of negative control wells were subtracted from the positive wells and results were expressed as spot-forming units (SFU) per 10^6^ PBMC. Responses were classified as positive if >3 times the mean of negative control wells and >50 SFU/10^6^ PBMC^[4]^.

**Supplementary Table 1. Amino acid positions under significant positive selective pressure in the HIV-2 and HIV-1 capsid.**Numbering based on HXB2 (M15390) and ROD (K03455) Gag positions. Only codons identified as significant sites by all algorithms run for each dataset are shown below.

|  | Codon | *dN-dS** | Normalized *dN-dS** | *P* value* | Amino acid (frequency)^ |
| --- | --- | --- | --- | --- | --- |
| HIV-2 | 254 | 5.28382 | 2.01982 | 0.000359 | A (0.56), P (0.37), Q (0.06), G (0.01) |
|  | 313 | 6.06039 | 1.91635 | 0.000196 | P (0.64), A (0.32), S (0.02), V (0.01), Q (0.01) |
|  |  |  |  |  |  |
| HIV-1 | 143 | 5.16769 | 4.68744 | 0.005756 | V (0.62), T (0.25), I (.13) |
|  | 146 | 5.12988 | 4.65304 | 0.003425 | S (0.6), P (0.31), A (0.09) |
|  | 173 | 3.5689 | 3.23723 | 0.020393 | T (0.58), S (0.4), A (0.02) |
|  | 215 | 7.0834 | 6.42512 | 0.000612 | V (0.56), T (0.29), I (0.09), Q (0.02), L (0.02), A (0.02) |
|  | 242 | 4.60437 | 4.17647 | 0.007989 | T (0.71), N (0.16) |
|  | 332 | 7.1192 | 5.57067 | 0.014335 | T (0.6), S (0.27), A (0.09), G (0.02), N (0.02) |

* Value from SLAC algorithm analysis

^ in n = 55 HIV-1 and n = 86 HIV-2 sequences

**Supplementary Table 2. Details of HIV-1 p24 codons under positive selective pressure in n = 55 sequences from the Caió cohort.** Only positions found to be statistically significant under all three algorithms (SLAC, FEL, FUBAR) are shown. Positions are numbered based on HIV-1 HXB2 Gag (M15390).

| **HXB2 Gag position** | **HXB2 amino acid** | **Known adapted polymorphisms at site and associated *HLA* class I alleles^[5]^*** | **Known epitopes with site included or in flanking region restricted by *HLA* class I alleles found in Caió**** |
| --- | --- | --- | --- |
| 143 | V | - | M**V**HQAISPR (A*3303) |
|  |  |  | GQM**V**HQAISPR (A*7401) |
|  |  |  | VQNLQGQM**V**(B*1302) |
|  |  |  | **V**HQAISPRTL (B*1510) |
|  |  |  | **V**HQAISPRTLNAW(B*3501) |
|  |  |  | QM**V**HQAISPRTLNAW (B*5801) |
| 146 | A | S (B13), P (B14), P (B57), A (C06), P (C08) | MVHQ**A**ISPR (A*3303) |
|  |  |  | **A**ISPRTLNAWV (A*6802) |
|  |  |  | GQMVHQ**A**ISPR (A*7401) |
|  |  |  | VQNLQGQMVHQ**A**(B*1302) |
|  |  |  | VHQ**A**ISPRTL (B*1510) |
|  |  |  | Q**A**ISPRTLNAW (B*3501) |
|  |  |  | **A**ISPRTLNAW(B*3502, B*3503, B*5301) |
|  |  |  | **A**ISPRTLNAW (B*5702, B*5703) |
|  |  |  | QMVHQ**A**ISPRTLNAW (B*5801) |
| 173 | S | I (B27), T (B57) | KAFSPEVIPMF**S** (A*0201, B*5801) |
|  |  |  | **S**ALSEGATPQDL (B*5301) |
|  |  |  | KVVEEKAFSPEVIPMF**S** (B*5703) |
| 215 | V | - | **V**HPVHAGPIA (B*3501) |
|  |  |  | AEWDR**V**HPV (B*5301) |
| 242 | T | N (B57/B58) | TTS**T**LQEQIA (A*6802) |
|  |  |  | TS**T**LQEQIGW (A*0201, B*5703, B*5801) |
| 332 | T | - | MTDTLLVQNANPDCK**T**IL (B*0801) |
|  |  |  | DCK**T**ILKAL (B*1503, B*5301) |
|  |  |  | NANPDCK**T**I (B*5101) |

*Analysis based on HIV-1 subtype B sequences only^[5]^.

** Based on epitope tables found at https://www.hiv.lanl.gov/content/immunology/maps/maps.html (data updated on 30.03.2017). Only potential epitopes associated with HLA class I alleles found in the Caió cohort are considered^[6]^. Amino acid of interest is highlighted in red. Where flanking regions are included, the putative epitope is underlined.

**Supplementary Table 3. High conservation within cytotoxic T-cell epitopes despite potent ELISpot responses to *HLA-B*14*, -*B*3501* and *-B*5301*-restricted epitopes in HIV-2-infected Caió participants.** Sites where amino acid variations are present within autologous sequences are underlined. These epitopes represent 3 of the 4 known cytotoxic T-cell epitopes in the HIV-2 capsid ^[7]^.

| **Participant** | **Peptide used** | **ELISpot response (SFU/10^6^ PBMCs)** | **Autologous sequence** |
| --- | --- | --- | --- |
| B14_1 | DRFYKSLRA | 215 | DRFYKSLRA |
| B14_2 |  | 1051 | DRFYKSLRA |
| B14_3 |  | 895 | DRFYKSLRA |
| B14_4 |  | 1020 | DRFYKSLRA |
| B3501_1 | NPVPVGNIY | 760 | NPVPVGNIY |
| B3501_2 |  | 350 | NPVPVGNIY |
| B3501_3 |  | 275 | NPVPVGNIY |
| B3501_4 |  | 1100 | NPVPVGNIY |
| B3501_5 |  | 1025 | NPVPVGSIY |
| B3501_6 |  | 2190 | NPIPVGNIY |
| B3501_7 |  | 155 | NPVPVGSIY |
| B5301_1 | TPYDINQML | 555 | TPYDINQML |
| B5301_2 |  | 20 | TPYDINQML |
| B5301_3 |  | 840 | TPYDINQML |
| B5301_4 |  | 1130 | TPYDINQML |
| B5301_5 |  | 3210 | TPYDINQML |
| B5301_6 |  | 2245 | TPYDINQML |
| B5301_7 |  | 155 | TPYDINQML |
| B5301_8 |  | 1100 | TPYDINQML |

**Supplementary Table 4. Clinical parameters and T-cell responses to a HIV-2 B*5801-resticted epitope (TW10-like) variants in HIV-2 infected *HLA-B*5801*-positive participants in Caió, Guinea-Bissau.** The codon positon identified as a possible *HLA-B*5801*-mediated escape mutation (E245D) is underlined.

|  | | | ELISpot responses  (SFU/10^6^ PBMCs) | | |  |
| --- | --- | --- | --- | --- | --- | --- |
| Participant | CD4 count (cells/µl) | HIV-2 VL (copies/ml) | TSTVEEQIQW (HIV-2 wild type) | TSTV**D**EQIQW (HIV-2 mutant) | TSTLQEQIGW  (HIV-1 TW10) | Autologous HIV-2 sequence* |
| B58_1 | 270 | <100 | 120 | 4400 | 0 | TSTV**D**EQIQW |
| B58_2 | 715 | <100 | 380 | 790 | - | TSTV**D**EQIQW |
| B58_3 | 670 | <100 | 110 | 250 | - | TSTV**D**EQIQW |
| B58_4 | 345 | 1085 | 145 | 845 | - | TSTV**D**EQIQW |
| B58_5 | 645 | 8340 | 445 | 2805 | - | TSTV**D**EQIQW |
| B58_6 | 1130 | <100 | 540 | 690 | - | TSTV**D**EQIQW |
| B58_7 | 940 | <100 | 935 | 135 | 0 | TSTVEEQIEW |
| B58_8 | 530 | 1589 | 845 | 0 | 0 | TSTVEEQIEW |
| B58_9 | 460 | 523 | 2160 | 2530 | - | TSTVEEQIQW |

VL = viral load, SFU = spot forming units, PBMCs = peripheral blood mononuclear cells.

* Autologous sequence generated from bulk PCR, therefore reflecting dominant sequence

**References**

1. [http://www.macrogen.com/](http://www.macrogen.com). In.

2. Delport W, Scheffler K, Botha G, Gravenor MB, Muse SV, Kosakovsky Pond SL. **CodonTest: modeling amino acid substitution preferences in coding sequences**. *PLoS Comput Biol* 2010; 6(8).

3. Kosakovsky Pond SL, Posada D, Gravenor MB, Woelk CH, Frost SD. **Automated phylogenetic detection of recombination using a genetic algorithm**. *Mol Biol Evol* 2006; 23(10):1891-1901.

4. Addo MM, Yu XG, Rathod A, Cohen D, Eldridge RL, Strick D, et al. **Comprehensive epitope analysis of human immunodeficiency virus type 1 (HIV-1)-specific T-cell responses directed against the entire expressed HIV-1 genome demonstrate broadly directed responses, but no correlation to viral load**. *J Virol* 2003; 77(3):2081-2092.

5. Brumme ZL, John M, Carlson JM, Brumme CJ, Chan D, Brockman MA, et al. **HLA-associated immune escape pathways in HIV-1 subtype B Gag, Pol and Nef proteins**. *PLoS One* 2009; 4(8):e6687.

6. Yindom LM, Leligdowicz A, Martin MP, Gao X, Qi Y, Zaman SM, et al. **Influence of HLA class I and HLA-KIR compound genotypes on HIV-2 infection and markers of disease progression in a Manjako community in West Africa**. *J Virol* 2010; 84(16):8202-8208.

7. Leligdowicz A, Yindom LM, Onyango C, Sarge-Njie R, Alabi A, Cotten M, et al. **Robust Gag-specific T cell responses characterize viremia control in HIV-2 infection**. *J Clin Invest* 2007; 117(10):3067-3074.
